# Changes in vocational college students’ mental health over two academic years: associations with family factors and co-curricular activities

**DOI:** 10.3389/fpubh.2026.1901932

**Published:** 2026-09-16

**Authors:** Zhaoying Ping, Wenshan Zhou

**Affiliations:** Anhui Zhong-Ao Institute of Technology, Hefei, China

**Keywords:** home-school collaboration, longitudinal changes in mental health, mental health, second classroom, vocational college students

## Abstract

**Background:**

Vocational college students face elevated risks of psychological distress due to academic, interpersonal and family stressors. However, longitudinal evidence tracking their two-year mental health shifts and relevant ecological correlates remains scarce. This study analyzed two-year mental health changes among vocational college students and examined correlations between family background variables and co-curricular activities (locally named “second classroom”).

**Methods:**

A two-year longitudinal survey was conducted with 1,277 students recruited from one vocational college in Anhui, China. Mental distress was measured via the Symptom Checklist-90 (SCL-90). Analyses included independent-samples t-tests, paired-samples t-tests, Pearson correlation, and multiple linear regression adjusted for baseline mental health scores.

**Results:**

Students exhibited statistically significant reductions in SCL-90 total scores at the two-year follow-up (*p* < 0.001), indicating improved psychological status. Family financial hardship and childhood left-behind experience correlated with higher levels of psychological distress. Co-curricular credit accumulation showed a weak negative correlation with post-test SCL-90 scores (*r* = −0.140, *p* < 0.001).

**Conclusion:**

Vocational students presented overall mental health improvements across two academic years. Family disadvantage correlates with higher psychological distress, while co-curricular participation only shows a mild negative correlation with follow-up mental symptoms with low explanatory power; co-curricular participation exhibits a potential supportive association with students’ mental wellbeing, yet it is merely one of numerous relevant factors shaping students’ psychological status. Optimizing structured co-curricular schemes can serve as one auxiliary mental health measure rather than a dominant intervention path.

## 1 Introduction

Higher vocational college students constitute a core youth group within China’s higher vocational education system, whose family backgrounds, academic pressures, career aspirations and self-perceived social identities differ markedly from those of general undergraduate students. This cohort commonly suffers from academic credential stigma, career confusion, employment pressure and persistent self-identity confusion. As the primary reserve of the national skilled workforce, dynamic shifts in their mental health exert far-reaching impacts on personal growth, career development, overall workforce quality and social stability, making research on this population’s psychological wellbeing of great practical significance.

Adolescent mental health has long been a core research topic worldwide, and numerous studies have explored the psychological status of vocational student cohorts. Cross-national systematic reviews and population-level surveys consistently demonstrate that vocational learners face elevated risks of psychological distress. Esch et al. (1) confirmed a bidirectional vicious cycle linking mental disorders and adolescent academic performance, whereby psychological troubles severely impair individuals’ psychological wellbeing and academic attainment. Mei et al. (2) pointed out that mental disorders represent the leading cause of disability for people aged 10 to 24, and the global high prevalence of youth mental health problems brings sustained adverse effects on academic performance, social functioning and long-term psychological development. A population-based survey conducted among Danish vocational school students further indicated suboptimal overall mental health in this group, with insufficient physical activity correlated with higher psychological risks (3).

From the perspective of positive psychology, international scoping reviews covering vocational training samples from multiple countries reveal that this cohort scores low on positive psychological resources such as subjective wellbeing and psychological resilience (4). A survey targeting 16–18-year-old vocational students in Malta found that 64.4% of respondents reported at least one psychological difficulty, indicating a severe detection rate of mental distress (5). In addition, the study conducted by Bucher et al. (6) indicated that only systematically organized outdoor co-curricular activities with full-time teacher supervision can exert observable positive effects on the mental health of vocational college students.

Domestic Chinese empirical studies have reached consistent conclusions: vocational college students exhibit higher detection rates of anxiety, depression, obsessive-compulsive symptoms and interpersonal sensitivity than general undergraduates, accompanied by prominent emotional disturbances and self-harm risks.

A review of existing literature reveals that vocational college students exhibit poor overall mental health with high detection rates of diverse psychological symptoms. Nevertheless, most previous studies rely on cross-sectional designs, with a scarcity of longitudinal surveys tracking two-year dynamic shifts in psychological status throughout vocational education. Drawing on Bronfenbrenner’s Ecological Systems Theory, this study only incorporates quantifiable indicators of the family microsystem and school microsystem as core predictors (12). This theoretical framework acts as a classification standard for variable screening. It also provides a foundational interpretative perspective to explain longitudinal shifts in psychological distress driven by proximal microsystem factors.

Co-curricular activities serve a core educational carrier in global vocational education research. International scholarship regards structured after-school programs as supplements to formal curricula that foster students’ sense of campus belonging, academic persistence and long-term psychological development (7). Multiple cross-national cross-sectional studies have verified that sustained participation in co-curricular activities correlates positively with adolescents’ mental wellbeing, and this association is more prominent among socioeconomically disadvantaged youth (8). Referred to as the “second classroom” in China, standardized credits-based co-curricular systems have been fully rolled out across domestic vocational colleges, covering ideological and moral education, physical and mental wellness training, cultural and artistic practice, innovation and entrepreneurship, as well as volunteer services. Such activities broaden students’ horizons and enrich their learning experiences, offsetting the deficiencies of formal classroom teaching in practical training and comprehensive literacy cultivation.

Against the above theoretical and research backdrop, the present study intends to map the mental health change of vocational college students throughout their campus years and examine the correlations between family background, co-curricular participation and students’ mental status, so as to provide targeted empirical evidence for the construction of home-school collaborative mental health intervention systems.

## 2 Research instruments and participants

### 2.1 Research instruments

Two standardized measurement indicators were adopted in this survey: the Chinese version of the SCL-90 scale was used to measure the level of psychological distress, and cumulative second classroom credits were applied to quantify students’ degree of participation in extracurricular practice.

#### 2.1.1 Symptom Checklist-90 (SCL-90)

This study adopted the Chinese version of the SCL-90 originally developed by Derogatis (9) and revised by Wang (10). Given the unstable replicability of the scale’s nine-factor structure among non-clinical adolescent samples in China, only the total score of the scale was used for all statistical analyses, and inferential tests were not conducted on subfactor scores. In accordance with China’s national normative screening criteria, respondents were judged to have potential psychological distress requiring further psychological assessment if any of the following three conditions were met: the total score exceeded 160, the number of positive items was more than 43, or the score of any single factor was ≥2. The scale presents favorable psychometric properties. The Cronbach’s *α* coefficient of the full scale was 0.97, the internal consistency coefficients of each subscale ranged from 0.77 to 0.90, and the 15-day test–retest reliability coefficients fell between 0.73 and 0.92. Its criterion correlation coefficients with mainstream clinical psychiatric scales ranged from 0.77 to 0.99. The total score can effectively distinguish healthy community residents from outpatients with mental disorders, which proves the scale is suitable for measuring the overall psychological distress level in this research.

#### 2.1.2 Second-classroom credit indicator

The second classroom, internationally referred to as co-curricular activities, is a nationwide unified credit-based extracurricular education system implemented in higher vocational colleges across China. It covers five activity categories: ideological and moral education, physical and mental wellness training, cultural and artistic practice, innovation and entrepreneurship, and social volunteer services. All activity credits are recorded into students’ comprehensive quality portfolios as a mandatory graduation requirement. It should be noted that accumulated credits only reflect the breadth and duration of students’ participation, and cannot measure the depth and subjective engagement quality of each single activity.

International co-curricular programs and China’s second classroom both serve as supplements to formal classroom teaching and aim to cultivate students’ practical abilities and sense of social responsibility. Nevertheless, the domestic second classroom system is supported by unified national policies, standardized institutional management and formal credit accreditation mechanisms, which can generate quantitative participation data applicable to longitudinal empirical analysis.

### 2.2 Research participants

Cluster sampling was used to recruit participants in this study. The target population covered all full-time freshmen of a higher vocational college in Anhui Province. Part-time students, exchange students and those with long-term off-campus absence were excluded to define a clear sampling frame. Intact freshman classes of all majors were taken as independent cluster units, and no class was split during data collection. Since this research adopted a single-institution design, stratified sampling and multi-site random sampling were not carried out, and a full census of all freshman clusters was implemented.

### 2.3 Data collection procedures

Prior to questionnaire distribution, researchers fully informed all participants of the survey objectives and data confidentiality rules. All raw data were de-identified to protect participants’ personal privacy, and all students took part in the survey voluntarily without any mandatory requirements.

Two standardized data collection waves were set in this research: the baseline pre-test was conducted at the early stage of freshman enrollment, and the follow-up post-test was carried out after students finished all core professional courses and before off-campus internships. Only participants who completed all core items in both rounds of questionnaires were included in the analytical dataset. Students who dropped out, transferred schools, or failed to finish the questionnaire in either wave were eliminated, and missing value imputation was not performed for participants lost to follow-up. After strict sample screening, a total of 1,277 valid participants with complete two-wave datasets were retained for all subsequent statistical analyses.

### 2.4 Statistical analysis

Descriptive statistics and paired-samples t-tests were used to compare the overall SCL-90 total scores and positive item counts between baseline pre-test and follow-up post-test, so as to describe the two-year changing of students’ psychological distress. Independent-samples *t*-tests were applied to compare differences in SCL-90 scores and cumulative second classroom credits across gender groups and groups with/without left-behind experience. Welch’s robust one-way ANOVA combined with Dunn’s post-hoc tests with adjusted *p*-values were adopted to examine intergroup disparities in mental health and co-curricular participation among three family economic strata. Pearson correlation analysis was conducted to test the bivariate correlation between second classroom credits and post-test SCL-90 indicators. All statistical analyses were run via SPSS. The analysis began with descriptive statistics and bivariate Pearson correlations. We then built multiple linear regression models to estimate how second-classroom credits predicted post-test psychological distress, with overall distress quantified using SCL-90 total scores. To eliminate bias arising from interindividual differences in baseline mental health, pre-test SCL-90 total scores were entered into the regression model as a primary covariate. We further adjusted for demographic factors, including gender, family financial status, and left-behind experience. Variance Inflation Factor (VIF) values were calculated to test for multicollinearity.

## 3 Research objectives

This study aims to depict the multi-dimensional mental health status of higher vocational college students. By comparing paired two-wave longitudinal data collected at baseline and follow-up, this study sorts out the temporal variations in students’ psychological status and relevant influencing factors, and further explores the relationship between co-curricular participation and changes in mental health. The general objective is to provide empirical evidence for optimizing mental health intervention and educational programs for vocational students.

## 4 Research results

### 4.1 Current mental health status of higher vocational students

According to the screening criteria of the Chinese national standardized norm for the SCL-90 scale, among the total valid paired sample of 1,277 students, 291 participants exceeded the cut-off value of 160 for the total score at the baseline pre-test, and 342 participants had more than 43 positive items. At the follow-up post-test, the number of cases with total score above 160 dropped to 213, and the number of participants with over 43 positive items decreased to 235. As shown in Table 1, the psychological distress positive screening rate declined visibly from baseline to follow-up. Additionally, the number of participants simultaneously meeting both abnormal thresholds (total score >160 and positive items >43) reduced from 271 cases at pre-test to 199 cases at post-test, which further verified the reduction in abnormal screening proportion (Table 1).

**Table 1:** Detailed breakdown of abnormal SCL-90 results.

Indicator	Sample size	Minimum value	Number of cases	Percentage (%)
SCL-90 Pre-test	1,277	SCL-90 Total Score>160	291	23
		SCL-90 Positive Items>43	342	27
		SCL-90 Total Score>160 & Positive Items>43	271	21
SCL-90 Post-test	1,277	SCL-90 Total Score>160	213	17
		SCL-90 Positive Items>43	235	18
		SCL-90 Total Score>160 & Positive Items>43	199	16

From a longitudinal research perspective, The statistical test yielded a statistically significant difference between baseline and follow-up total scores (*p* < 0.001). Descriptive statistics indicated that the mean SCL-90 total score at baseline was 134.69, which was statistically significantly higher than the follow-up mean score of 122.05 (Table 2). In accordance with the scale scoring rule, a higher total score corresponds to a higher level of psychological distress. Such a statistically significant reduction in average total scores across the two time points preliminarily reflects an overall improvement in students’ mental health conditions over the academic years; their psychological status prior to off-campus internship was statistically better than the status at the initial enrollment stage.

**Table 2:** Detailed breakdown of SCL-90 pre-test and post-test differences.

Indicator	Paired (Mean ± SD)		Difference (pre-test-post-test)	*t*	*p*
	SCL-90 total score pre-test	SCL-90 total score post-test			
SCL-90 total score	134.69 ± 44.72	122.05 ± 49.10	12.64	8.699	*p* < 0.001

^*^p < 0.05, ^**^p < 0.01, ^***^p < 0.001.

### 4.2 Differences in mental health among different groups

#### 4.2.1 Analysis of gender differences in mental health status

Between-gender disparities in SCL-90 total scores among higher vocational college students were analyzed based on the two-wave survey data (Table 3). The test results revealed a statistically significant gender difference in baseline pre-test SCL-90 total scores (*p* < 0.001). Female students obtained a significantly higher average total score (139.44 ± 45.10) than male students (125.20 ± 42.45), which reflected that female freshmen presented severer self-perceived psychological distress symptoms at enrollment. In the follow-up post-test stage, however, the independent-samples *t*-test demonstrated no statistically significant gender discrepancy in SCL-90 total scores (*p* = 0.803). Such contrasting findings preliminarily indicate that the predictive effect of gender on students’ psychological distress levels attenuated throughout their academic study period.

**Table 3:** Mental health status by gender.

Indicator	Gender (Mean ± SD)		*t*	*p*
	Male (*n* = 426)	Female (*n* = 851)		
SCL-90 Total Score (Pre-test)	125.20 ± 42.45	139.44 ± 45.10	−5.534	*p* < 0.001
SCL-90 Total Score (Post-test)	122.55 ± 53.29	121.79 ± 46.89	0.249	0.803

*p < 0.05, **p < 0.01, ***p < 0.001.

#### 4.2.2 Differences in mental health status by left-behind growth experience

SCL-90 subscale scores were analyzed between students with and without childhood left-being experience across the two survey waves. (Table 4). The statistical outcomes demonstrated that left-behind experience produced statistically significant between-group differences in SCL-90 total score and the count of positive items at both baseline pre-test and follow-up post-test stages (all *p* < 0.05). It can be concluded that early left-behind growth experience has a persistent adverse impact on students’ psychological conditions from enrollment to the period prior to off-campus internship.

**Table 4:** Mental health status with and without experience of left-behind.

Indicator	Did you experience being left behind during your growth process? (Mean ± SD)		*t*	*p*
	Yes (*n* = 704)	No (*n* = 573)		
SCL-90 total score (Pre-test)	137.43 ± 46.12	131.32 ± 42.74	2.434	0.015*
SCL-90 positive items Pre-test	29.92 ± 24.11	27.03 ± 23.50	2.151	0.032*
SCL-90 total score (Post-test)	124.77 ± 51.16	118.70 ± 46.26	2.222	0.026*
SCL-90 positive items Post-test	21.89 ± 27.52	18.65 ± 25.66	2.172	0.030*

^*^p < 0.05, ^**^p < 0.01, ^***^p < 0.001.

Specifically, participants who reported left-behind experience obtained higher mean values on all four indicators: pre-test total score (137.43 ± 46.12), pre-test positive items (29.92 ± 24.11), post-test total score (124.77 ± 51.16), post-test positive items (21.89 ± 27.52), which were statistically significantly greater than the corresponding scores of non-left-behind students (131.32 ± 42.74, 27.03 ± 23.50, 118.70 ± 46.26, 18.65 ± 25.66). Such findings reflect that early left-behind experience is correlated with elevated psychological distress symptoms in vocational college students.

#### 4.2.3 Mental health differences by family economic pressure levels

Welch’s robust one-way ANOVA was adopted for intergroup comparison of SCL-90 total scores across three family economic pressure groups, and Dunn’s test with adjusted *p*-values was further applied for pairwise post-hoc comparisons. Family economic hardship was measured by students’ self-reported financial situation (Table 5).

**Table 5:** Mental health status of students under different family economic pressures.

Indicator	What is your family’s financial situation? (Mean ± SD)			*F*	*p*
	Severe hardship (*n* = 160)	Moderate hardship (*n* = 654)	No hardship (*n* = 463)		
SCL-90 Total Score (Pre-test)	144.26 ± 51.77	135.01 ± 44.05	130.94 ± 42.58	5.341	0.005**
SCL-90 Total Score (Post-test)	134.10 ± 64.51	122.66 ± 48.17	117.01 ± 43.25	7.379	0.001**

^*^p < 0.05, ^**^p < 0.01, ^***^p < 0.001.

The main effect of family economic pressure was statistically significant for SCL-90 total scores at both baseline pre-test and follow-up post-test assessments (pre-test: *F* = 5.341, *p* = 0.005**; post-test: *F* = 7.379, *p* = 0.001**). Descriptive statistics showed a clear gradient trend: the no-hardship group yielded the lowest average scores (pre-test:130.94 ± 42.58; post-test:117.01 ± 43.25), followed by the moderate-hardship group (pre-test:135.01 ± 44.05; post-test:122.66 ± 48.17), while the severe-hardship group obtained the highest symptom scores in both waves (pre-test:144.26 ± 51.77; post-test:134.10 ± 64.51). Higher SCL-90 total scores reflected more severe psychological distress symptoms.

Subsequent Dunn’s pairwise comparisons (Table 6) further examined intergroup differences driving the significant main effect. At pre-test, the comparison between the severe-hardship group and the no-hardship group showed no statistically significant difference (*p* = 0.051). Similarly, the post-test contrast between these two groups also did not reach statistical significance (*p* = 0.077). All pairwise comparisons involving the moderate-hardship group against the other two groups yielded non-significant results at the *α* = 0.05 level across both measurement waves.

**Table 6:** Pairwise comparison results of SCL-90 total score via Dunn’s *t*-test (adjusted *p*-values).

Indicator	Group (I)	Group (J)	Median (I)	Median (J)	Difference (I-J)	*p*
SCL-90 total score (Pre-test)	Severe hardship	Moderate hardship	131.500	120.000	11.500	0.487
	Severe hardship	No hardship	131.500	118.000	13.500	0.051
	Moderate hardship	No hardship	120.000	118.000	2.000	0.349
SCL-90 total score (Post-test)	Severe hardship	Moderate hardship	104.000	100.000	4.000	1.000
	Severe hardship	No hardship	104.000	98.000	6.000	0.077
	Moderate hardship	No hardship	100.000	98.000	2.000	0.134

*p < 0.05, ^**^p < 0.01, ^***^p < 0.001.

### 4.3 Differences in participation in co-curricular activities

#### 4.3.1 Gender differences in co-curricular activity participation

A higher credits value of the second classroom merely indicates that students participate in more extracurricular activities; it does not necessarily reflect a higher intensity of engagement or higher quality of participation in each activity. Through data analysis, gender differences in the accumulated credits of the Co-curricular Activities (Second Classroom)among higher vocational college students were examined (Table 7). The results revealed a highly significant gender difference in co-curricular credits (*t* = −6.951, *p* < 0.001). Female students obtained a significantly higher average credit score (116.93, SD = 33.19, *n* = 851) than male students (101.49, SD = 39.36, *n* = 426). Within this single institutional sample, gender presented a statistical association with the level of co-curricular engagement; females demonstrated greater initiative to participate in various second classroom activities compared with males.

**Table 7:** Gender participation in extracurricular activities.

Indicator	Your gender? (Mean ± SD)		*t*	*p*
	Male (*n* = 426)	Female (*n* = 851)		
Second-classroom credits	101.49 ± 39.36	116.93 ± 33.19	−6.951	*p* < 0.001

*p < 0.05, **p < 0.01, ***p < 0.001.

#### 4.3.2 Differences in co-curricular activity participation by left-behind experience

Through data analysis, the accumulated Co-curricular Activities (second classroom) credits of students with and without childhood left-behind experience were examined (Table 8). The independent-samples t-test indicated no statistically significant between-group difference in total co-curricular credits (*t* = 0.031, *p* = 0.975 > 0.05). Descriptive statistics demonstrated nearly identical average credits scores for the two groups: 111.81 ± 34.83 (*n* = 704) for students with left-behind experience and 111.75 ± 37.61 (*n* = 573). Within the sample of this single vocational college, no statistical association was observed between left-behind growth experience and the degree of students’ participation in second classroom activities.

**Table 8:** Participation in second-classroom activities by students with and without left-behind experience.

Indicator	Did you experience being left behind during your growth process? (Mean ± SD)		*t*	*p*
	Yes (*n* = 704)	No (*n* = 573)		
Second classroom credits	111.81 ± 34.83	111.75 ± 37.61	0.031	0.975

^*^p < 0.05, ^**^p < 0.01, ^***^p < 0.001.

#### 4.3.3 Co-curricular engagement differences across different family economic pressure groups

Welch’s robust one-way analysis of variance (ANOVA) together with Dunn-adjusted post-hoc pairwise comparisons were adopted to examine group disparities in co-curricular credits among vocational college students with varying degrees of family financial pressure. Family economic conditions were measured by students’ self-reported subjective evaluation. The omnibus test demonstrated a highly statistically significant overall difference in total co-curricular credits among the three groups (Table 9). Descriptive statistics indicated that students with moderate family financial hardship obtained the highest average co-curricular credits, followed by students with severe family hardship, while students with no financial hardship had the lowest average score.

**Table 9:** The participation of students in second-classroom activities under different family economic pressure.

Indicator	What is your family’s financial situation? (Mean ± SD)			*F*	*p*
	Severe hardship (*n* = 160)	Moderate hardship (*n* = 654)	No hardship (*n* = 463)		
Second classroom credits	110.65 ± 36.79	115.47 ± 35.86	106.95 ± 35.66	7.722	*p* < 0.001

^*^p < 0.05, ^**^p < 0.01, ^***^p < 0.001.

Post-hoc Dunn’s pairwise comparisons (Table 10) pinpointed the exact source of the overall group difference: a highly significant difference was only detected between the moderate financial hardship group and the no hardship group. No statistically meaningful disparities in co-curricular credits were observed between severe hardship and moderate hardship groups, as well as between severe hardship and no hardship groups.

**Table 10:** Pairwise comparison results of second-classroom credits via Dunn’s *t*-test (adjusted p-values).

Indicator	Group (I)	Group (J)	Median (I)	Median (J)	Difference (I-J)	*p*
Second classroom credits	Severe hardship	Moderate hardship	111.150	115.050	−3.900	0.237
	Severe hardship	No hardship	111.150	109.500	1.650	1.000
	Moderate hardship	No hardship	115.050	109.500	5.550	*p* < 0.001

^*^p < 0.05, ^**^p < 0.01, ^***^p < 0.001.

### 4.4 Relationship between second classroom participation and mental health

Pearson correlation analysis was firstly adopted to separately test the correlations between co-curricular credits and two mental health indicators, namely the post-test total score of SCL-90 and the post-test count of SCL-90 positive items. The post-test SCL-90 total score had a statistically significant negative correlation with co-curricular credits at the 0.001 level. Such negative correlation revealed that higher co-curricular engagement was associated with a lower post-test SCL-90 total score, which suggested that second co-curricular activities might exert a potential supportive effect on students’ mental health. In addition, co-curricular credits were also significantly negatively correlated with the number of post-test SCL-90 positive items (Table 11).

**Table 11:** Analysis of correlation between participation in second-classroom activities and mental health level.

Variable	Statistic	SCL-90 total score post-test	SCL-90 positive items post-test
Second classroom credits	Correlation coefficient	−0.140**	−0.123**
	*p* value	*p* < 0.001	*p* < 0.001
	Sample size	1,277	1,277

^*^p < 0.05, ^**^p < 0.01, ^***^p < 0.001.

On the basis of correlation results, multiple linear regression analysis was carried out, with the post-test SCL-90 total score set as the dependent variable and co-curricular credits treated as the core independent variable.

To eliminate the confounding bias caused by the inherent difference in initial mental health level. Pre-test SCL-90 total score was incorporated into the model as a key confounding covariate. Meanwhile, gender, family financial status and left-behind experience were included as demographic controlled covariates based on 1,277 valid samples (Table 12).

**Table 12:** Multiple linear regression results for post-test SCL-90 total score adjusted for baseline SCL-90 score, demographic and family covariates.

Variable	Nonstandardized coefficient		Standardized coefficient	*t*	*p*	Collinearity diagnostics	
	*B*	Standard error	*Beta*			VIF	Tolerance
Constant	107.936	8.525	-	12.662	*p* < 0.001	-	-
Second classroom credits	−0.189	0.035	−0.139	−5.344	*p* < 0.001	1.049	0.954
SCL-90 total score (Pre-test)	0.426	0.028	0.388	15.016	*p* < 0.001	1.036	0.965
Gender	−3.843	2.734	−0.037	−1.406	*p* = 0.16	1.069	0.935
Family financial situation	−5.640	1.934	−0.075	−2.916	*p* < 0.01	1.039	0.963
Left-behind experience situation	−2.159	2.546	−0.022	−0.848	*p* = 0.397	1.032	0.969
*R* ^2^	0.18						
Adjust *R*^2^	0.177						
*F*	*F*(5,1,271) = 55.883, *p* < 0.001						
D-W value	2.062						

Dependent variable = SCL total score post-test (*n* = 1,277).

*p < 0.05, **p < 0.01, ^***^p < 0.001.

The overall regression model reached high statistical significance *F* (5,1,271) = 55.883, *p* < 0.001and the adjusted *R*^2^ value indicated that the whole set of predictors could explain 17.7% of the variance in students’ post-test psychological distress scores. The Durbin-Watson statistic was 2.062, which excluded residual serial autocorrelation; the VIF values of all predictors were lower than 1.10, verifying that no obvious multicollinearity existed in the regression equation.

For the core predictor, co-curricular credits still showed a statistically significant negative predictive effect on the post-test SCL-90 total score after fully controlling for baseline pre-test SCL-90 level and demographic covariates (*β* = −0.139,*p* < 0.001)Higher co-curricular engagement was linked to lower follow-up psychological distress. Among the controlled variables, family financial status exerted a significant negative predictive influence on mental health indicators (*β* = −0.075, *p* < 0.01), meaning students with better family economic conditions tended to obtain lower psychological distress scores. However, gender and left-behind experience did not produce statistically significant predictive effects on the dependent variable (*p* > 0.05 for both).

## 5 Discussion and reflections on research results

### 5.1 Limitations of the present study and interpretation constraint

First, It is critical to clarify that this study only includes two measurement waves (baseline and two-year follow-up). We merely examined group-level pre-post mean differences in psychological distress, and no modeling of individual mental health trajectories was performed within our analytical framework. In this manuscript, the term “longitudinal changes” solely refers to the group-level pre-post variations we analyzed across the two waves of data.

Second, all cross-sectional subgroup comparisons (including gender and left-behind experience differences) only identify correlational relationships between grouping variables and psychological distress, and this study cannot confirm strict causal links between these factors and students’ mental health conditions.

Third, although the overall mental health level of participants showed a significant improvement from baseline to follow-up, such longitudinal variation merely reflects sequential temporal changes over students’ vocational education period. Restricted by the single-group pre-post longitudinal design without blank control groups, we cannot attribute the overall improvement of psychological status to co-curricular (Second Classroom) activities or other school educational interventions. Further quasi-experimental research with control groups is required to systematically explore the independent influencing mechanism of co-curricular participation on mental health.

Fourth, the multiple linear regression model in this study only included a small set of demographic and family predictors, while numerous critical ecological factors closely linked to vocational students’ mental health (such as peer relationships, campus atmosphere, career anxiety, teacher-student interaction and employment pressure) were not incorporated into the analytical framework. Given the incomplete inclusion of explanatory variables, the model exhibits constrained explanatory capacity. All research conclusions and derived practical enlightenment must be interpreted under the premise of insufficient variable inclusion, and excessive generalization of the research findings should be avoided.

### 5.2 The overall mental health level of higher vocational college students improves during school years

Longitudinal follow-up data in the present study revealed that vocational college students demonstrated an upward change in overall mental health across the two academic years from admission to the eve of off-campus internship. Paired comparison of pre-test and post-test scores on the SCL-90 scale demonstrated that the mean pre-test total score (134.69) was statistically significantly higher than the mean post-test total score (122.05). Statistically significant declines were observed in both the total score and the count of positive items on the scale, which reflected a notable drop in the percentage of students experiencing psychological distress.

This longitudinal outcome aligns with abundant domestic empirical studies focusing on vocational college students’ mental health, which further corroborates that the work implemented in higher vocational institutions may have a potentially beneficial effect on students’ mental health.

### 5.3 The potential supportive association between co-curricular participation and students’ mental wellbeing

Empirical analytical results revealed a statistically significant but weak negative longitudinal correlation between co-curricular credits and follow-up psychological distress (*β* = −0.139, *p* < 0.001) after fully adjusting for the key confounding covariate of pre-test SCL-90 total score, together with demographic covariates including gender, family financial status and left-behind experience (Table 12). The multiple regression model achieved an adjusted *R*^2^ of 0.177, indicating that the whole set of predictors collectively explained 17.7% of the variance in post-test psychological distress. Notably, the strong predictive contribution originated mainly from baseline pre-test mental health level (*β* = 0.388, *p* < 0.001).

From the perspective of Ecological Systems Theory, participation in the second classroom acts as a supportive resource within the school microsystem. Nevertheless, the present study reveals that engagement in the second classroom merely acts as an auxiliary protective factor rather than a core determinant of students’subsequent psychological status. Specifically, higher second-classroom credits corresponded to lower post-test SCL-90 total scores. Collinearity diagnostics indicated that all variance inflation factor (VIF) values were below 1.10, which ruled out multicollinearity bias and confirmed the robustness and credibility of the analytical findings.

The intrinsic psychological mechanism accounting for this correlation can be interpreted via a dual-pathway theoretical framework. On the one hand, co-curricular programs involving group teamwork and volunteer services broaden students’social connection networks, alleviate feelings of loneliness and interpersonal sensitivity, and fulfill their psychological needs for belongingness and social engagement. On the other hand, chances for skill presentation and practical innovation within these activities deliver positive behavioral feedback, strengthen students’self-efficacy and self-esteem, which may explain the observed negative correlation with adverse emotional symptoms.

### 5.4 The non-negligible influence of family factors on students mental health

From the perspective of Ecological Systems Theory, family economic status and left-behind experience constitute stable risk-related factors within the family microsystem. A disadvantaged family environment may exert long-term adverse associations with the mental health development of vocational college students. The mental health disparities associated with family financial pressure remained stable across the two waves of longitudinal surveys.

From the perspective of family functioning, SCL-90 data showed that students without left-behind experience had lower average total scores than those with left-behind backgrounds. Independent-samples t-tests identified significant group differences (*p* < 0.05) in SCL-90 total scores at both testing waves, demonstrating higher psychological distress among students who experienced left-behind growth.

In terms of family financial circumstances, descriptive SCL-90 statistics indicated that students without financial burdens obtained lower average symptom scores than those experiencing moderate or severe economic strain. One-way ANOVA detected consistent significant intergroup differences across both survey waves (*p* < 0.01). Although pairwise comparisons with adjusted *p*-values failed to reach statistical significance, the data presented a clear economic gradient effect: higher levels of family financial pressure corresponded to elevated psychological distress scores. This suggests that the adverse impacts of financial hardship on mental health are progressive and cumulative, rather than only manifesting abruptly among students in extreme poverty.

On the whole, the family environment constitutes a fundamental cornerstone of adolescents’ psychological development and may exert far-reaching associations with their long-term mental health development. To a certain degree, household financial constraints limit students’ accessibility to daily living resources and social practice opportunities. Prolonged parental separation during childhood may impede the establishment of secure attachment, and such adverse developmental impacts frequently carry forward into vocational college education. Importantly, left-behind experience does not dampen college students’ willingness and proactive motivation to join co-curricular activities. In response, higher vocational colleges ought to continuously diversify the content and organizational forms of co-curricular activities, and Schools can set up diversified activity platforms for left-behind students to expand social networks and display personal strengths; co-curricular participation exhibits a potential supportive association with students’ mental wellbeing.

### 5.5 Constructing a mental health safety net through home-school partnership

From the perspective of ecological systems theory, individual development does not occur in isolation, and interconnections and interactions exist across distinct microsystems. In the present study, family and school environments represent core components of the microsystem, and their interplay exerts tangible impacts on the mental health of vocational college students. The vocational college period constitutes a critical developmental transition from adolescence to young adulthood, during which students encounter a multitude of stressors and challenges spanning academics, interpersonal relationships, emotional regulation, and career planning. Higher vocational institutions serve as the primary setting for students’ learning and daily life, equipped with standardized, systematic mental health education frameworks alongside diversified resources for social practice and campus cultural activities. Regular participation in school-organized second-classroom activities alleviates psychological maladjustment and delivers a buffering effect for students lacking emotional support from their families.

Nevertheless, school-based resources cannot fully substitute the protective functions of the family environment. The family constitutes the core primary environment for individuals’ psychological development. The quality of family relationships, household emotional atmosphere, and parental rearing styles profoundly shape adolescents’ personality traits and emotional adaptation patterns. As illustrated in Elffers’ (11) Dutch doctoral research, adverse early family upbringing diminishes students’ motivation to engage in extracurricular practice, and this inhibitory effect is moderated significantly by gender. It follows that family contexts exert direct impacts on students’ engagement in campus activities, which underscores the necessity of promoting home-school partnership to build multi-layered support systems for students’ development.

## 6 Conclusion

This two-wave longitudinal study of vocational college students yielded three core findings. First, participants’ overall psychological distress levels decreased significantly over two academic years; disadvantaged family financial conditions and left-behind experience were persistently correlated with poorer mental health across both surveys. Second, after adjusting for baseline mental status and demographic covariates, second-classroom credits exhibited a statistically significant yet weak negative predictive effect on follow-up psychological distress, indicating that co-curricular participation merely acts as a limited school-based supportive resource rather than a dominant protective factor. Third, the mandatory credit requirement for graduation in Chinese vocational colleges diluted the inhibitory effect of adverse family backgrounds on extracurricular engagement, and gender and left-behind experience showed no independent predictive power for subsequent mental distress.

Conditional practical implications can be drawn based on the correlational evidence of this observational study. Vocational colleges may optimize structured second-classroom programs as an auxiliary measure to ease students’ psychological distress, though such activities cannot replace family emotional support. Schools should establish targeted home-school collaborative support systems focusing on students with financial hardship and left-behind experience to offset risks originating from the family microsystem. Meanwhile, the unified credit system narrows participation gaps among students from diverse family backgrounds, and diversified activity forms are recommended to strengthen its mild mental health benefits.

## Data Availability

Due to the protection of participants’ privacy, the anonymized dataset is available from the corresponding author upon reasonable request.
